# Moving to a healthier city? An analysis from China's internal population migration

**DOI:** 10.3389/fpubh.2023.1132908

**Published:** 2023-02-13

**Authors:** Ping Gao, Wei Qi, Sheng He Liu, Zhen Liu, Ze Han Pan

**Affiliations:** ^1^Institute of Geographic Sciences and Natural Resources Research, Chinese Academy of Sciences (CAS), Beijing, China; ^2^Institute for Population Research, Fudan University, Shanghai, China

**Keywords:** environmental health, intercity population migration, spatial pattern, influencing factor, China

## Abstract

A healthy urban environment is considered as an important issue for the amenity and equity of migrants. China has one of the largest internal population movements in the world, and the environmental health of its migrants becomes a growing concern. Based on the 1‰ microdata from the 2015 1% population sample survey, this study uses the spatial visualization and spatial econometric interaction model to reveal intercity population migration patterns and the role of environmental health in China. The results are as follows. First, the main direction of population migration is toward economically developed high class cities, especially the eastern coast where the intercity population migration is most active. However, these major destinations are not necessarily the healthiest areas for the environment. Second, environmentally friendly cities are mainly located in the southern region. Among them, the areas with less serious atmospheric pollution are mainly distributed in the south, climate comfort zones are mainly located in the southeastern region, but areas with more urban green space are mainly distributed in the northwestern region. Third, compared with socioeconomic factors, environmental health factors have not yet become a major driver of population migration. Migrants often place higher value on income than on environmental health. The government should focus not only on the public service wellbeing of migrant workers, but also on their environmental health vulnerability.

## 1. Introduction

Throughout the history of human development, population migration and population distribution have always tended to favor areas with suitable environments, which are suitable for human survival and also improve human health and life expectancy (1–3). With the onset of the industrialization period, socioeconomic factors became important drivers of changing population migration patterns. Migrants have a stronger need for job opportunities, income levels and social and public services (4–6). However, in recent years, with the emergence of climate change, environmental pollution and ecological damage, more and more studies have focused on the relationship between population migration and environmental health (7–9). On the one hand, migrants, especially migrant workers, are considered as a vulnerable group in the city. Concerns have been raised about the equity of migrant health in urban environments. On the other hand, as the level of socio-economic development has increased, a healthy urban environment has become an important consideration for migrants in making migration decisions. Some new terms, including amenity migration and lifestyle migration, are emerging (10, 11). Therefore, the role of migrants should not be overlooked when discussing the urban environment and health issues. However, existing studies have focused more on forced migration due to environmental changes but less on active migration. Moreover, no consensus conclusion has been reached on the effect of environmental health on population migration.

To compensate for these shortcomings, this study selects China, the country with the highest population and the most prominent scale of internal migration, to explore in-depth the impact of environmental health factors on population migration. Since the reform and opening up of China in 1978, a massive transfer of surplus rural labor to the cities has occurred. Some of the migrants have settled in cities and become urban residents. However, a large number of migrants remain who are only workers in the cities, and this group is known as the floating population in China (12). According to the data of the seventh national census, China's current floating population is 376 million, and the proportion of the floating population in the total urban population has reached 41.6%. Unlike local urban residents, the floating population does not have complete urban social security and is often separated from other members of the family (13). The government has actively introduced policies to strongly improve the social welfare level of the floating population. However, compared with social vulnerability, the environmental health equity vulnerability of floating population has long been neglected. Chinese cities face many environmental challenges, including air pollution, green space shortages and water scarcity (14). Although most of the floating population treat the destinations as a place of work, and many are even still willing to return and settle in their hometown, they are permanently exposed to the environment of the destination cities. Focusing on the environmental health of the floating population is a topic of health equity for those who move and the long-term development of a healthy city (15).

This study focuses on whether urban environmental health factors influence migrants' migration decision and destination choice. The spatial patterns of intercity migration flows and the strength of their environmental health factors are investigated, taking China as an example. The second part gives a systematic literature review in which we summarize the research frontiers on the patterns and drivers of population migration, with a focus on the impact of environmental health factors. The third section presents the research methodology of this study. The fourth part is the structure of the analysis, including the spatial pattern of intercity population migration in China, the spatial pattern of environmental health factors in Chinese cities, and the mechanism of the effect of environmental health factors on population migration. The fifth part is the discussion. The sixth part concludes.

## 2. Literature review

### 2.1. Patterns of population migration

Numerous theoretical and empirical studies have shown that migrants tend to move to larger commercial or industrial centers, and the great body of migrants only proceed a short distance. In developing countries, population migration is dominated by rural-urban migration, while in developed countries, reverse urbanization has occurred. Inter- and intra-city migration will become mainstream during the developed society period. The laws of migration also pointed out that migration from counties surrounding big cities such as London and Manchester leaves gaps in the rural population, which are subsequently filled by migrants from more remote districts, thus net migration flows were upward alone the urban hierarchy, and the biggest inflow for any level is that for its exchanges with units of the next smaller size (16, 17). This step migration is still predominant in today's developing countries. However, the US's hierarchical migration is strongly contrasting, many of the major movements are flows down the urban hierarchy (18), which has become the norm in some developed countries.

Unlike most countries, population migration in China is characterized by the hukou system, which is the nation's household registration institution (19). Hukou is a type of permit that allows migrants to enjoy social welfare as local citizens do. In other words, a migrant who lacks the hukou in the destination cannot be an honest citizen like those residents who possess the hukou (20). Owing to the hukou, a unique feature of population migration in China is its two-track system, consisting of permanent migration and temporary migration (21). The former refers to movements that are accompanied by hukou change, while the latter refers to movements that are not associated with hukou change (22). Temporary migrants are known as floating population or no-hukou migrants; they are mainly rural-urban individuals and cannot enjoy the benefits and rights of permanent migrants and local residents in destination cities, such as social securities, health care and education opportunities (23). Recently, no-hukou migrants have become the main body of urbanization and citizenization and deserve more attention. Thus, the population migration in this study mainly refers to no-hukou migration.

Since the reform and opening up, China adopted a coastal development strategy which allowed some coastal areas to develop first, resulting in a reversal in the direction of migration: more migrants moved from the western and central to the eastern, from inland to coastal areas (24–26). The pattern of population migration in China is relatively concentrated. Three major developed urban agglomerations in the Yangtze River Delta, Pearl River Delta and Beijing–Tianjin–Hebei were the main centers of migration destination, while the less developed central regions were the main migration sources (27). Some scholars believed that this spatial polarization was continuously strengthened, indicating gainers gaining more and losers losing more population from net migration (28). However, some others argued that this polarization began to decline in the 21st century, with a trend of decentralization and landization (29, 30). Recently, many interior areas have undergone a tide of industrialization and received many labor-intensive industries transferring from coastal regions, potentially heralding a decrease in eastward migration and an increase in backflow in the coming decades (31). Evidence also shows that settling permanently in the destination city is difficult for the vast floating population; thus, most of them adopt a circular flow pattern to travel between the origin and destination cities (32).

China has a large number of intra-provincial instead of inter-provincial no-hukou migrants, accounting for 66.8 and 33.2% in 2020, respectively (33). However, due to the limitation of data acquisition, the studies on migration patterns in China are mainly limited to the inter-provincial scale (34, 35). The intra-provincial scale, especially the inter-city level, which may have a greater impact on urbanization and regional development, has gained little attention (36). Recently, some studies have realized the importance of scale and tried to study migration patterns at the prefecture-level city scale (37–39), but they mainly used big data to study short-term daily mobility, which is essentially different from population migration. Migration, rather than mobility, has a greater impact on the urban system and urbanization process and is thus more worthy of study. Only in recent years has the literature begun to examine intercity population migration in China. For example, Liu et al. (40) studied the stability and change in China's geography of intercity migration based on a complex network approach, finding that the migration network is stable but also becomes significantly dispersed due to the increasing short-distance and intra-provincial migration. Mu et al. (41) revealed an emerging reversal from a predominantly upward pattern (e.g., most of the net flows move to high-level cities) to a downward one (e.g., from super-large/extra-large cities to large cities).

### 2.2. Drivers of population migration and environmental health factors

Traditional migration theory generally believed that economic factors play a decisive role in population migration. The laws of migration considered migration as an inseparable part of economic development, and the major cause of migration is economic (16). Neoclassical theory considered migration as a function of geographical differences in the supply and demand for labor. The resulting wage differentials encourage workers to move from low-wage, labor-surplus areas to high-wage, labor-scarce areas (42). Migration network theory believed that population migration is a path-dependent process; already settled migrants often act as a “bridgehead” (43), reducing the risks and costs of subsequent migration and settlement by providing information, organizing travel, finding jobs and housing and assisting in adaptation to a new environment, thereby promoting more migration. According to the gravity law and the radiation model (44), population size and distance are also the main factors influencing population migration.

Environmental migration is an issue that is often considered as new or a part of future trends. In fact, it is a long-standing phenomenon (45). Environmental factors ranked highly in the first systematic theories of migration. In Ravenstein's “the laws of migration,” he mentioned unattractive climate (46). Semple (47) pointed out that the search for better land, milder climate and easier conditions of living starts many a movement of people which, in view of their purpose, necessarily leads them into an environment sharply contrasted to their original habitat. However, with the onset of the industrialization period, socioeconomic factors became important drivers of changing population migration patterns; references to the environment as an explanatory factor gradually disappeared from the migration literature. Theoretical publications, such as migration transition theory, neoclassical theory and ecological models, gave the most central place to socioeconomic factors but did not mention environmental factors (17, 48, 49). This is because with economic development and technological advances, the influence of nature on population migration and distribution continued to diminish. Petersen (50) even believed that environmental migration as a primitive form of migration is bound to decline as human beings gradually increase their control over their environment.

However, with the emergence of climate change, environmental pollution and ecological damage, more and more studies have refocused on the relationship between population migration and environmental health. These studies mostly started with amenity migration, focusing on natural amenities such as climate and air quality. For example, Graves (51, 52) argued that under the assumption that individual utility of labor is uniform, the differences in labor wage between regions are compensations for amenity, thus, migration is essentially based on the need for regional amenity rather than wage differences. Gottlieb (53) argued that urban amenity is often seen as a commodity, with non-tradable and place-specific characteristics, and people choose residential migration to satisfy the demand for such goods. In recent years, as developed countries enter the post-industrial era, lifestyle migration, residential tourism and retirement migration have become the focus of academic attention (54). These migrations are mostly White residents of the Global North moving part- or full-time to “their” paradise in the Global South, not motivated primarily by economic need but by a desire to consume a particular set of amenities critical to an imagined recreational lifestyle unavailable or unaffordable in their home country. The U.S. migration-pattern regime also shows that many of the major movements in the system of domestic migration are flowing down the urban hierarchy (18), one of the main reasons is the desire for a healthy environment for some migrants as they change over the life cycle.

In China, numerous empirical studies confirm economic incentives and socio-cultural conditions, such as differences in wages, living standards, job opportunities, public facilities and services, are important determinants of migration decisions and destination choices (55). However, migrants are not only economic people pursuing economic benefits but also social people pursuing better quality of life, that is, when the physiological or material needs of migrants are satisfied, they will breed the demand for high quality of life. With the transition of young, high-quality migrants and the family-oriented migration mode, the literature has begun to focus on environmentally driven amenity migration studies, and environmental health begins to become a concern as an influencing factor for population migration in China. For instance, Cao et al. (27) found that the natural environment gradually became an attractive factor that migrants considered. Liu and Yu (56) found that there is a significant and negative effect of air pollution on migrants' interest in settling down. Liu and Shen (31) suggested that China's skilled people prioritize their career prospects over the quality of life; climatic amenities exert a strong influence on skilled migration but have a positive effect on less-skilled migration at the origin and no effect at the destination.

In terms of methodology, the gravity law and radiation model are the prevailing framework to predict population movement (44). The size of migration flows mainly depend on the push and pull factors of the origin and destination and the distance attenuation effect but has nothing to do with other migration flows, which ignore the spatial dependence between migration flows, and cannot disclose the multilateral spillover mechanism in the migration process (57). On this basis, Griffith and Jones (58) proposed the idea of using the spatial lag of the dependent variable or error term to capture spatial dependence. Lesage and Pace (59) extended the gravity model by introducing the spatial lag of explained variable and proposing a spatial econometric interaction model, which provides an effective analytical tool for quantitatively analyzing the “multilateral effect” of migration flows. In addition, some scholars tried to use a spatial filtering model to filter out network autocorrelation (60). These methods have effectively reduced the deviation of parameter estimates and significantly improved the model's accuracy, but they often filter out some meaningful information, such as spatial spillover effects.

## 3. Methodology

### 3.1. Study area

**Figure 1:**
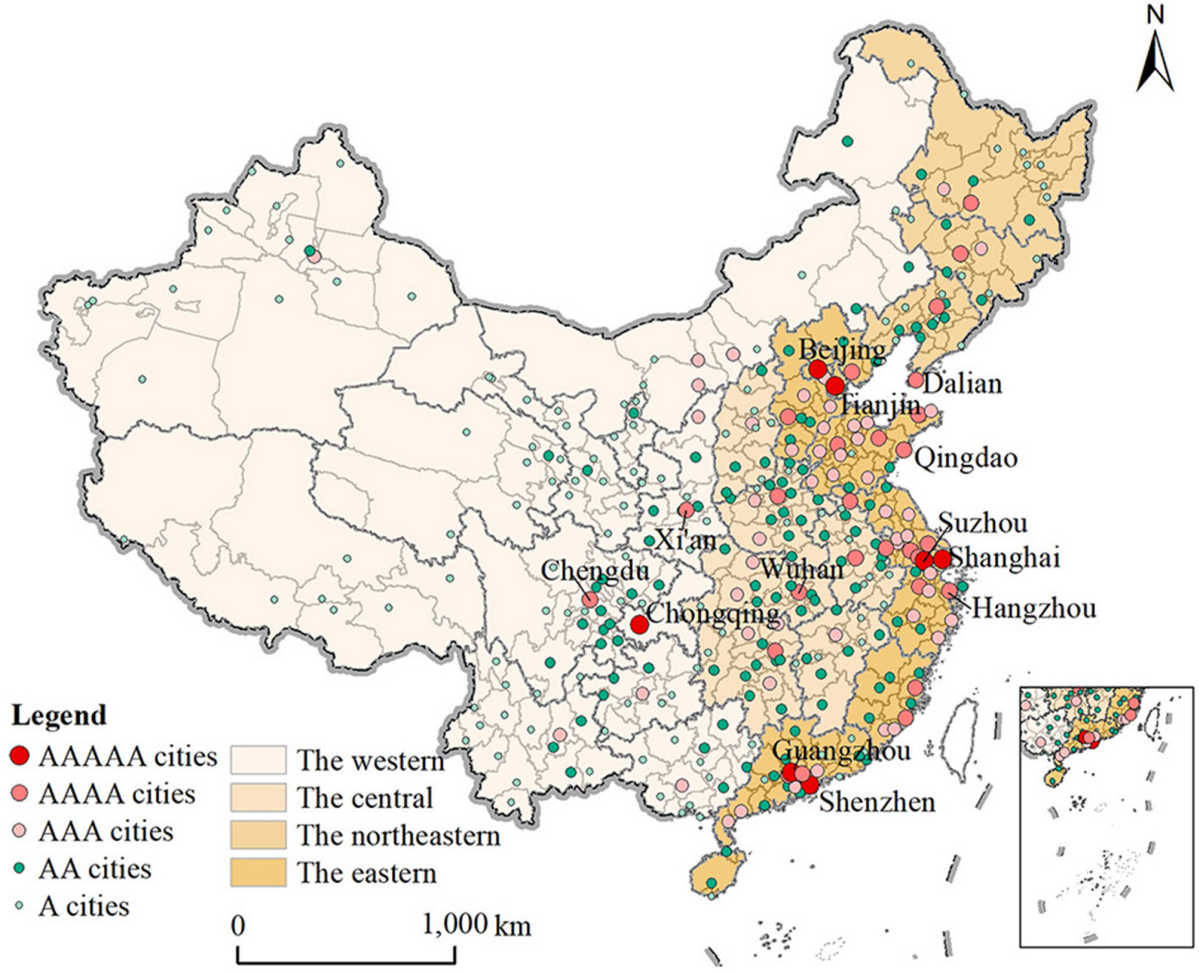
The geographic location of four economic regions and the city hierarchy classification.

This study focuses on the population migration flows between cities in China. The term “city” in this study refers to 341 prefecture-level or above administrative units, comprising 4 municipalities (Beijing, Shanghai, Tianjin and Chongqing), 15 sub-provincial cities, 17 general provincial capital cities, 300 general prefecture-level cities and 5 provincial-controlled divisions. It is officially designated administrative territory, not physical territory. Except for Qingdao, Dalian, Ningbo, Xiamen and Shenzhen, the remaining sub-provincial cities are also provincial capital cities. Taiwan and Sansha in Hainan Province are not included due to unavailable data. According to the National Bureau of Statistics, these cities are grouped into four economic regions: eastern, central, western and northeastern (Figure 1).

This study also uses the natural breaks slice method to divide cities into five levels of hierarchy based on the economic scale of each city, that is, A, AA, AAA, AAAA and AAAAA cities, representing low, lower-middle, medium, upper-middle and high income cities, respectively. The economic scale can reflect a certain level of development, and the use of economic scale to classify city hierarchy can not only examine the direction and internal structure of hierarchical migration, but also reflect the relationship between the structure of hierarchical migration and the level of development from the side. The number of A, AA, AAA, AAAA and AAAAA cities was 7, 27, 49, 119, and 138, respectively, presenting a pyramid structure.

### 3.2. Models and variables

#### 3.2.1. Spatial econometric interaction model

Lesage and Pace (59) summarized the spatial dependence relationship between population flows into three types. The first is “destination-based” spatial dependence, that is, the flows from origin A to destination B will change with the flows from the same origin A to the surrounding areas of destination B. The second type is “origin-based” spatial dependence, that is, the flows from origin A to destination B will change with the flows from the surrounding areas of origin A to the same destination B. Third is “origin-to-destination-based” or “flow-based” spatial dependence, that is, the flows from origin A to destination B will change with the flows from surrounding areas of origin A to the surrounding areas of destination B. On this basis, three network weight matrices (*W*_*d*_, *W*_*o*_, *W*_*w*_) are used to construct the spatial lag form of the dependent variable (*W*_*d*_*y*, *W*_*o*_*y*, *W*_*w*_*y*), to form the spatial autoregressive form of the gravity model, that is, the spatial OD model, also called the spatial econometric interaction model. Its general expressions are as follows:


$$\begin{array}{l} y=\rho_{d}W_{d}y+\rho_{o}W_{o}y+\rho_{w}W_{w}y+\alpha \tau_{N}+{X^{{}^{\prime }}}_{d}\beta_{d}+{X^{{}^{\prime }}}_{o}\beta_{o}+\gamma g+\epsilon \end{array}$$

The model contains *n*^2^ = *N* pairs of OD migration flows, *y* represents the *N*×1 column vector of intercity migration flow. *W*_*d*_*y*, *W*_*o*_*y*, *W*_*w*_*y* are the “destination-based”, “origin-based” and “origin-to-destination-based” dependent variables spatial lag, representing the weighted average flows to the destination neighbors, from the origin neighbors, and from the origin neighbors to the destination neighbors, respectively. ρ_*d*_, ρ_*o*_, ρ_*w*_ represent the corresponding spatial dependence parameters, respectively, reflecting the intensity of three types of spatial autocorrelation effect. When spatial autocorrelation is not considered (ρ_*d*_ = ρ_*o*_ = ρ_*w*_ = 0), the spatial OD model becomes the gravity model. τ_*N*_ is a *N*×1 column vector whose all elements are 1. α is the constant term coefficient of τ_*N*_. *X* is the *n*×*k* explanatory variable matrix, repeating *X n* times to obtain an *N*×*k* destination explanatory variable matrix ${X^{{}^{\prime }}}_{d}$ and repeating each row of *X n* times to obtain an *N*×*k* origin explanatory variable matrix ${X^{{}^{\prime }}}_{o}$. β_*d*_, β_*o*_ are the corresponding influence coefficients. *g* is the *N*×1 distance matrix between cities. γ is the distance friction coefficient. ϵ is an *N*×1 error perturbation term, which obeys the standard normal distribution.

#### 3.2.2. Explanatory variables of intercity population migration

The environment is closely related to people's health, and a suitable environment is beneficial to human survival and health. In this study, the environmental factors affecting human health are defined as environmental health factors, among which the influence of natural environment is particularly prominent. Therefore, the term “environment” in this study mainly refers to the natural environment, namely, the total of various inartificial and artificially modified natural factors that affect human survival and development. It follows that environmental health factors are environmental factors related to population health, are part of environmental conditions/factors, and sometimes it can also refer to healthy environmental factors. This study selects air quality, climate comfort and green space as proxy variables for environmental health factors. The air quality index and climate comfort index are two negative indicators. The larger the value, the worse the air quality and climate comfort.

In addition, socio-economic factors as well as gravity factors are also important factors influencing intercity population migration; they are included in the model as control variables. Among them, economic level, wage income, job opportunity and living cost are selected as proxy variables for economic factors. Social network, education level, medical level and cultural service are selected as proxy variables for social factors. Population size, spatial distance and temporal distance are selected as proxy variables for gravity factors. The same explanatory variables are selected for each city as origin and destination; “_o” and “_d” are added after the variables to distinguish the two roles. The variable descriptions, expected effects and data sources are shown in Table 1.

**Table 1:** The variables system of influencing factors of inter-city population migration.

**Variables**	**Descriptions**	**Expected effect**	
		***_o***	***_d***
**(1) Environmental health factors**			
Air quality	Air quality index (AQI)^a^	+	-
Climate comfort	Climate comfort index (THI)^b^	+	-
Green space	Per capita park green area (PARK, km^2^/person)^c^	-	+
**(2) Economic factors**			
Economic level	Per capita GDP (PGDP, ten thousand Yuan)^c^	-	+
Wage income	The average wages of employees (WAGE, ten thousand Yuan)^c^	-	+
Job opportunity	The proportion of employees in secondary and tertiary industries (PJOB, %)^c^	-	+
Living cost	The house price-to-income ratio (COSH, %)^c^	+	-
**(3) Social factors**			
Social network	Migration stock (SOC)^d^	+	
Education level	The number of teachers per primary and secondary students (EDU)^c^	-	+
Medical level	The number of tertiary hospitals (HEAL)^c^	-	+
Cultural service	The number of books in the public library per 100 people (CUL)^c^	-	+
**(4) Gravity factors**			
Population size	The size of permanent population (POP, million people)^e^	+	+
Spatial distance	The straight-line distance between two cities (DIS, km)^f^	-	
Temporal distance	The shortest time spent on the closest route and fastest means of transportation (TDIS, h)^f^	-	

Sources:

^a^https://www.aqistudy.cn/historydata/.

^b^THI = |T-0.55(1-RH)(T-58)-65|, T is temperature(°F), RH is relative humidity(%), temperature and humidity data are from the Resource and Environment Science and Data Center.

^c^China city statistical yearbook in 2016.

^d^SOC = the number of migrants from city *i* to city *j*/the total number of migrants from city *i*.

^e^Tabulation on the 2015 1% population sample survey.

^f^Calculated by Network Analyst Tools in ArcGIS based on traffic network data, which are from Practical Atlas of China.

### 3.3. Data sources

The data used in this study are mainly aggregated intercity population migration flows, including size and direction. Intercity population migration refers to the migration process in which the “current residence” and “domicile place” are not in the same city for more than half a year. These data can be gathered from the 1‰ micro-database of the 2015 1% population sample survey (thereafter, 2015 microdata), which includes 1.37 million personal records, accounting for 1‰ of the total population in China. After weighting, the data of each region has been converted according to the national uniform sampling ratio to ensure the samples' composition represents that of the actual population. Thus, the data can be directly compared. In addition, the data sources of influencing factors are detailed in Table 1.

## 4. Results

### 4.1. Patterns of intercity population migration in China

#### 4.1.1. Spatial heterogeneity of migration flows

**Figure 2:**
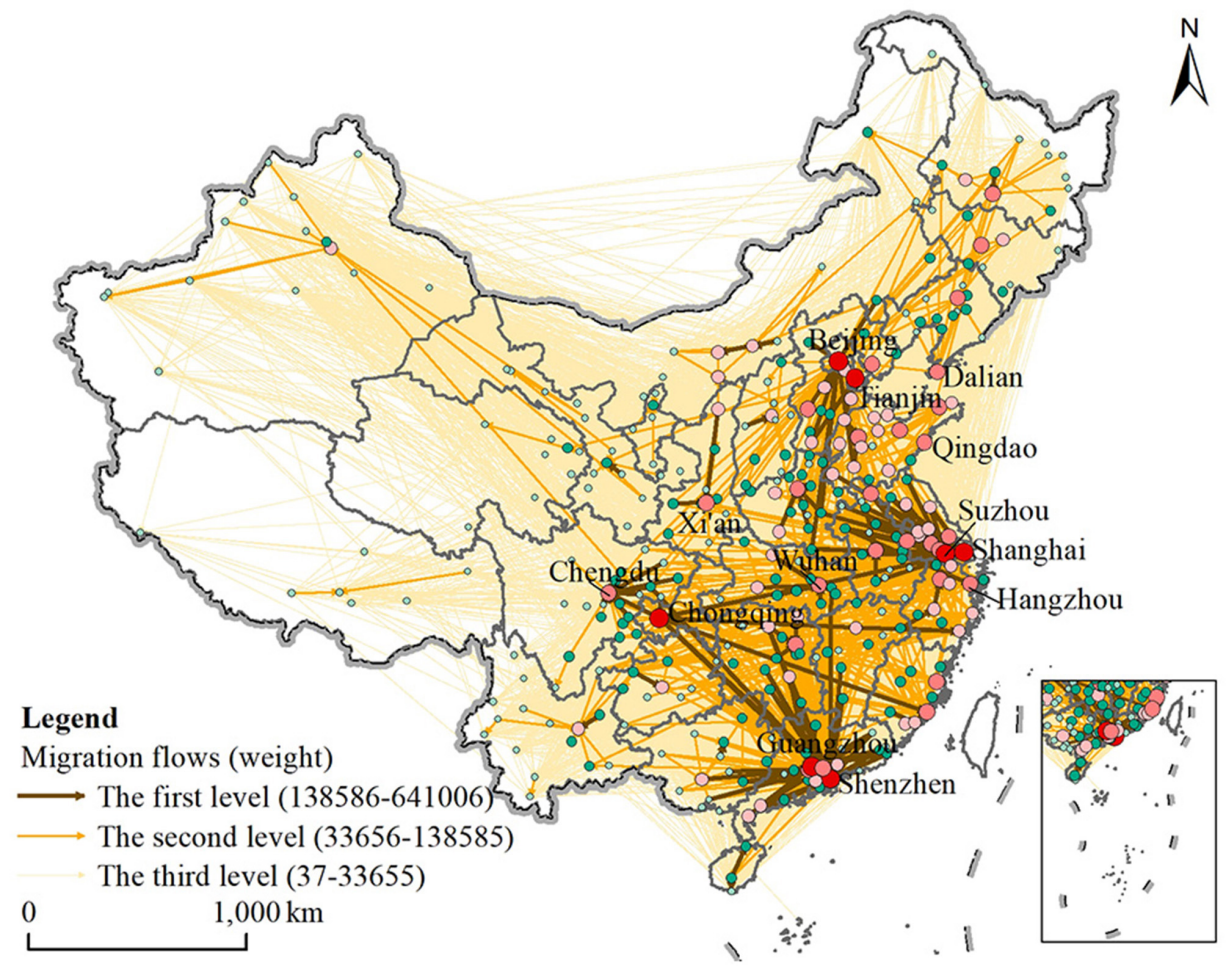
The spatial pattern of intercity migration flows at different levels in China.

There are 15,472 intercity migration flows in China, carrying a total of 153 million intercity floating population, with an average of 9,900 people per flow and a maximum flow of 641,000 people. In space, intercity migration flows show obvious spatial heterogeneity (Figure 2). Firstly, there are 138 first-level flows, accounting for 0.89% of total flows. These stronger migration flows basically distribute in the southeast half of China, especially between the core cities and their surrounding cities in three major developed coastal urban agglomerations: the Yangtze River Delta, the Pearl River Delta and the Beijing–Tianjin–Heibei regions. Next are the two major developed inland urban agglomerations, the Chengdu–Chongqing City Cluster and the Triangle of Central China, whose core cities, Chengdu and Wuhan, are also important destination cities that attract a large number of migrants within the province. Chongqing is an important outflow city, showing long-distance migration to developed eastern coastal cities, such as Dongguan, Quanzhou, Wenzhou and Shanghai.

Then, there are 782 second-level flows, accounting for 5.05% of total flows. The core cities in three major developed coastal urban agglomerations continue to expand their hinterland range, covering most of the southeastern half of China. The population gathering capacity of sub-provincial cities and general provincial capital cities are gradually prominent, such as Jinan, Qingdao, Xi'an, Kunming, Xiamen and Harbin. Flows in the northwestern half of China began to appear, such as the flows between Urumqi, which is the provincial capital city of Xinjiang, and its surrounding cities. Finally, there are 14,551 third-level connections, accounting for 94.06% of total flows, implying that intercity migration flows are dominated by weak intensity flows. These flows still mainly occur in the southeastern half of China, but the coverage has expanded to the national scope.

It follows that intercity population migration flows still tend to move from less developed cities to developed cities and has formed a “three big and two small” polycentric spatial pattern. Three big centers refer to the regions of Beijing–Tianjin–Hebei, the Yangtze River Delta and the Pearl River Delta, while the two small centers mainly refer to the Chengdu–Chongqing City Cluster and the Triangle of Central China. Unlike the inter-provincial migration pattern from the central and western to the eastern, the regional distribution of intercity migration flows is mainly within the eastern cities, with a total of 36.58 million people (Table 2), followed by the migration from the central and the western to the eastern, reaching 35.65 and 21.92, respectively. This is because the majority of high-intensity migration flows are mainly within the three major eastern urban agglomerations, clustering from peripheral cities to a few core cities, such as Beijing, Shanghai, Shenzhen, Dongguan and Guangzhou.

**Table 2:** The statistics of intercity migration flows between four economic regions (million).

**Regions**	**The eastern**	**The central**	**The western**	**The northeastern**
The eastern	36.58	3.00	3.26	0.52
The central	35.65	15.74	3.23	0.44
The western	21.92	1.68	21.85	0.41
The northeastern	3.45	0.27	0.56	4.54

#### 4.1.2. Hierarchical migration pattern

**Figure 3:**
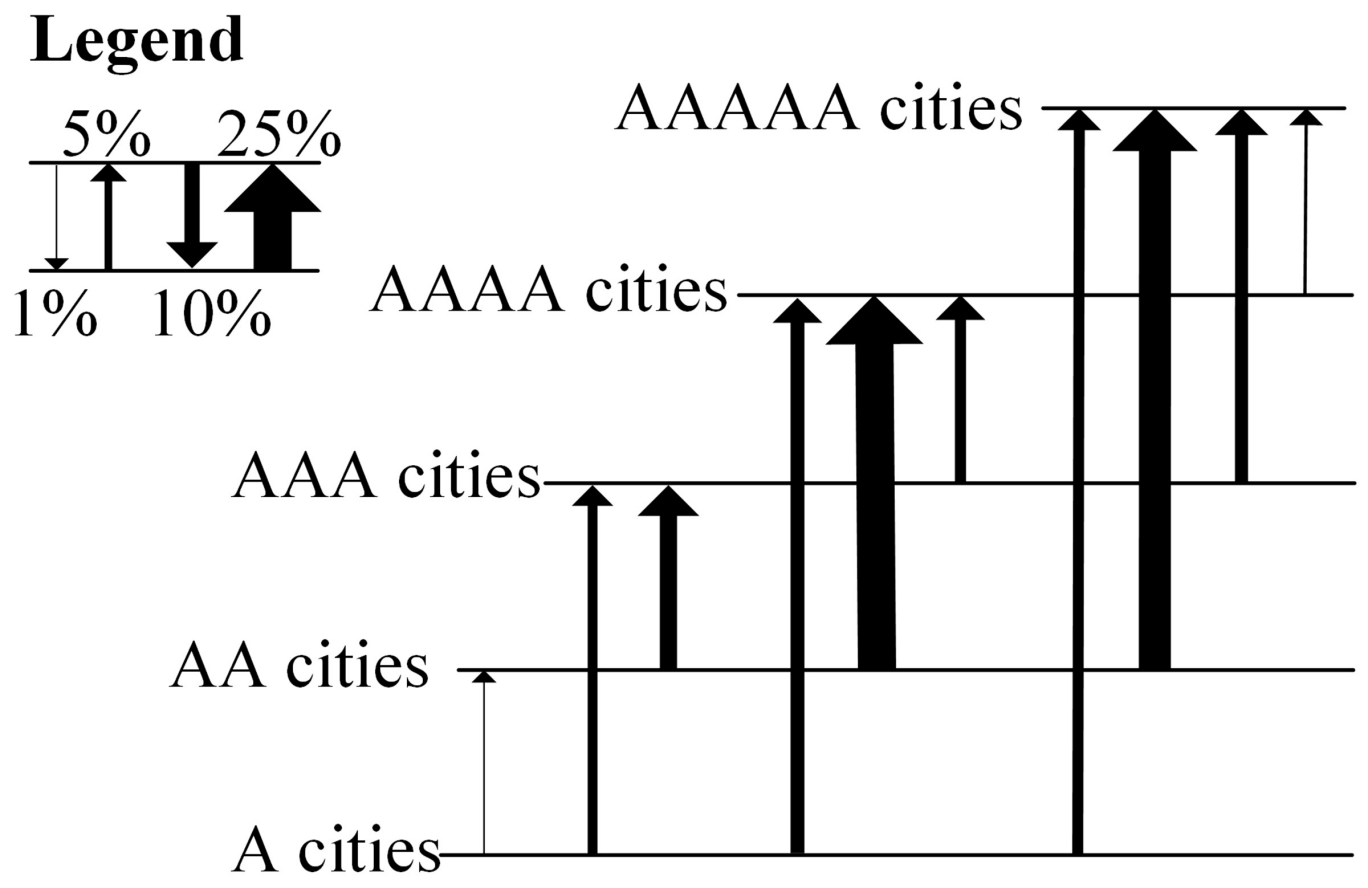
The net migration flows up the city hierarchy. The arrow of the line indicates the direction of net migration, and the width of line indicates the percentage of total net migration between all levels of the hierarchy.

Intercity population migration has the characteristics of hierarchical migration, that is, intercity net migration flows go up the development-based city hierarchy (Figure 3), with net migration from lower-income cities to higher-income cities and lower-income cities attracting migrants from poorer cities. This result agrees with Ravenstein's migration system, where labor gaps left by people leaving semi-peripheral areas to central areas are filled by migrants from even more peripheral areas, which is also called replacement migration. However, unlike Ravenstein's migration system where the largest net flows are the step migration between adjacent levels of hierarchy, namely, the biggest inflow for any level is its exchanges with units of the next lower level, intercity population migration in China is a type of cross-level jump migration. Here, the largest net flows are the jump migration from AA cities to AAAA cities, accounting for 25.2%, followed by migration from AA cities to AAAAA and AAA cities, accounting for 21.1 and 11.4%, respectively.

It follows that intercity population migration still conforms to the economic law of migration, moving up the urban economic hierarchy, but it is a jump migration from lower-medium-income cities to higher-income cities. It is noteworthy that most intercity net migration flows neither come from the poorest cities nor from the poorest segments of the population, which can be explained by the aspiration-capabilities model (61). Migration involves significant costs and risks. Although people in the poorest cities have high migration aspirations, their migration capabilities sometimes can not afford these migration costs and risks, such as funds for travel, housing and living expenses. However, development in low-income cities boosts migration because improvements in income, infrastructure and education typically increase people's capabilities and aspirations to migrate. Lower-middle income cities therefore tend to be the most migratory, and migrants predominantly come from relatively better-off sections of origin populations.

### 4.2. Spatial differences in environmental health in Chinese cities

**Figure 4:**
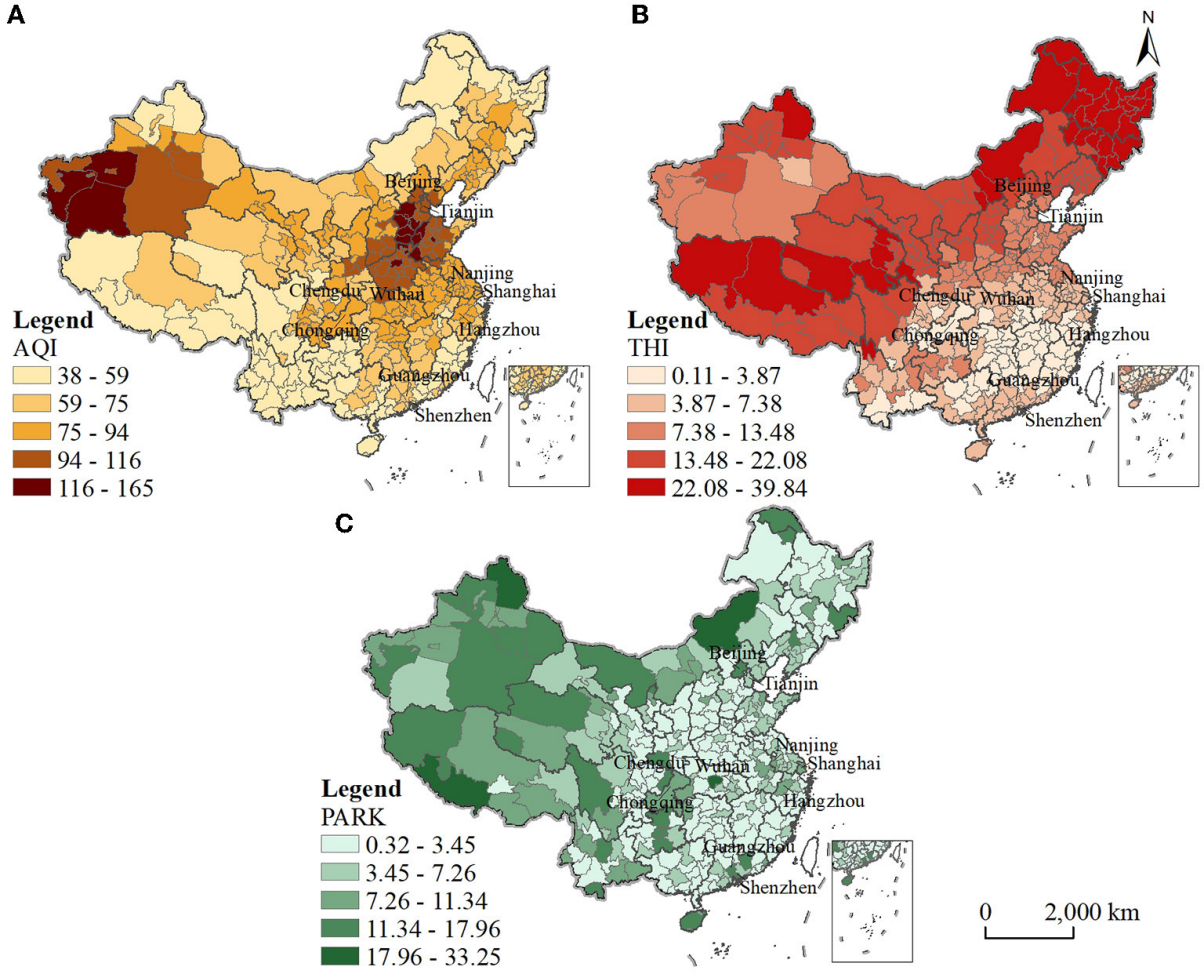
The spatial pattern of three environmental health factors. **(A)** Air quality index. **(B)** Climate comfort index. **(C)** Per capita park green area.

Environmentally friendly cities are mainly located in the southern region. Here, the air quality index (AQI) shows a spatial pattern of “high in the north and low in the south” (Figure 4A), which means that the air quality condition in the south is better than that in the north. On the one hand, the high AQI areas are mainly in the northern regions: the Beijing–Tianjin–Hebei region and its neighboring Shandong Province and Henan Province, as well as the central and western regions of the Xinjiang Autonomous Region. Their industrialization level is relatively high and the industrial structure is relatively heavy, generating a large amount of waste gas, slag and wastewater, resulting in serious environmental pollution problems. However, core cities in these regions, such as Beijing and Tianjin are important destinations for migrants. On the other hand, the low AQI areas are mainly located in the south, especially in the southwest, where the industrial development is mainly commercial and service industries, with a relatively light industrial structure, high precipitation and high forest coverage. The air quality conditions are good.

The climate comfort index (THI) shows a spatial pattern of “high in the northwest and low in the southeast” (Figure 4B), which means that the climate comfort condition in the southeast is higher than that in the northwest. On the one hand, the high THI areas are concentrated in the three northeastern provinces, Qinghai–Tibet Plateau region and Xinjiang Autonomous Region, Inner Mongolia Autonomous Region and Gansu Province in the northwest. They are relatively unsuitable for human habitation due to their location at higher latitudes or high altitudes and extremely low temperatures in winter. On the other hand, the low THI areas are mainly located in the southern region, especially the cities in the central Hunan Province, Jiangxi Province and the eastern Fujian Province; their temperature and humidity are relatively moderate in the four seasons and more suitable for human habitation. However, the majority of cities in these provinces are important origins for migrants.

The per capita park green area (PARK) shows a spatial pattern of “high in the northwest and low in the southeast” (Figure 4C), meaning that the ecological environment in the northwest is relatively better than that in the southeast. On the one hand, the high PARK areas are mainly located in the Qinghai–Tibet Plateau region and the Xinjiang and Inner Mongolia Autonomous Regions in the northwest. These cities are economically underdeveloped but have high forest coverage and sparse populations, resulting in larger per capita green space areas and good ecological environments. On the other hand, the low PARK areas are mainly located in the central, eastern and northeastern regions. These cities have relatively high levels of urbanization, and the development of the urban built environment has crowded out a large amount of ecological space, together with a large population, leading to a low per capita park green area. Some cities even face the dilemma of lacking ecological public space.

### 4.3. Impact of environmental health factors on intercity population migration in China

#### 4.3.1. Environmental health factors

The impact of AQI on intercity population migration is not in line with expectations. In general, if the AQI increases by 1%, its inflows will significantly increase by 0.04%, and its outflows will significantly reduce by 0.37% (Table 3), indicating that the more serious air pollution in the city is, the more migrants it attracts and the less local people it moves out. The reason may be related to the level of industrialization. The spatial distribution of AQI shows that important migration destinations, such as Beijing and Tianjin, tend to have higher levels of industrialization but relatively serious air pollution. By contrast, most of the southern cities with lower levels of industrialization but better air quality are important sources of migrants, leading to statistically more migrants moving into cities with more serious air pollution, and from cities with better air quality. Thus, air quality has minimal effect on intercity population migration.

**Table 3:** The estimation results of gravity model and spatial econometric interaction model.

**Log (variables)**	**Gravity model**		**Spatial econometric interaction model**	
	**Coefficient**	**t value**	**Coefficient**	**t value**
Const	7.3840^***^	46.824	6.8599^***^	119.434
lAQI_o	−0.4041^***^	−27.284	−0.3579^***^	−66.477
lAQI_d	−0.0049	−0.312	0.0343^***^	6.050
lTHI_o	−0.8559^***^	35.463	−0.8640^***^	98.799
lTHI_d	−0.1136^***^	4.391	−0.0799^***^	8.481
lPARK_o	0.0171^***^	3.196	0.0116^***^	6.018
lPARK_d	0.0010	0.186	0.0005	0.268
lPGDP_o	−0.0510^***^	−5.487	−0.0385^***^	−11.503
lPGDP_d	0.0392^***^	4.272	0.0149^***^	4.462
lWAGE_o	−0.1904^***^	−9.806	−0.1880^***^	−10.438
lWAGE_d	0.0351^**^	2.324	0.0347^**^	2.435
lJOB_o	−0.1849^***^	−20.613	−0.1810^***^	−56.142
lJOB_d	0.0218^**^	2.389	0.0120^***^	3.628
lCOSH_o	−0.1176^***^	−9.183	−0.1156^***^	−25.086
lCOSH_d	−0.0263^**^	−2.332	−0.0506^***^	−12.392
lSOC	0.9226^***^	336.681	0.9030^***^	874.790
lEDU_o	0.2228^***^	16.001	0.2225^***^	44.438
lEDU_d	0.02287	1.533	0.0428^***^	7.960
lHEAL_o	−0.0407^***^	−6.489	−0.0427^***^	−18.927
lHEAL_d	−0.0002	−0.037	0.0173^***^	7.967
lCUL_o	−0.3051^***^	−51.523	−0.2917^***^	−136.200
lCUL_d	0.0137^**^	2.311	−0.0085^***^	−3.916
lPOP_o	0.9200^***^	139.589	0.8822^***^	355.467
lPOP_d	0.0180^***^	2.925	0.0149^***^	6.629
lDIS	−0.0087	−1.205	0.0219^***^	8.207
lTDIS	−0.0491^***^	−6.562	−0.0326^***^	−12.013
ρ_*d*_	-	-	0.0252^***^	40.581
ρ_*o*_	-	-	0.0146^***^	26.498
ρ_*w*_	-	-	−0.0029^***^	−3.353
AIC	12,633.53	-	12,034.56	-

^***^ and

^**^ indicate passing the significance tests of 1% and 5%, respectively.

THI has a significant pulling effect on the inter-city inflows, but the impact on the outflows does not meet expectations. Generally, if the THI increases by 1%, its inflows will significantly reduce by 0.08%, but its outflows will significantly reduce by 0.86%, showing obvious asymmetry. This means that the more comfortable the urban climate is, the more floating population it attracts but the more population outflow it promotes. This radiation effect is far greater than the attractive effect. This is mainly because the vast majority of cities located in the southeast with a comfortable climate are also important sources of emigration, leading to a statistically more comfortable climate with more emigrants, reflecting perspective that climate is not an important consideration in the migration decision of migrants. However, the attraction of climate comfort in the choice of migration destination is beginning to emerge.

The impact of PARK on intercity population migration is not in line with expectations. In general, if the PARK increases by 1%, its inflows will increase by 0.0005% but are not significant, and the outflows will increase by 0.01%. That is, a good ecological environment does not attract more floating population but will push more local people to move out. This is mainly because the cities with higher PARK are mostly small and medium-sized cities in the northwestern half of China with lower population density, plot ratio, and economic development level. They are often accompanied by more emigration, resulting in a better ecological environment with statistically more emigrants. Cities with lower PARK are mostly located in the southwest half of China, including both important emigration and immigration cities, resulting in statistical insignificance, reflecting the perspective that green ecological space is also not an important factor for intercity population migration.

#### 4.3.2. Other influencing factors

The impact of per capita GDP, the proportion of employees in secondary and tertiary industries, and the average wages of employees are in line with expectations. Generally, if these three indicators increase by 1%, their inflows will increase significantly by 0.02, 0.03, and 0.01%, respectively, and their outflows will decrease significantly by 0.04, 0.19, and 0.18%, respectively. This means that migrants still tend to move to (from) cities with higher (lower) economic development levels, more (fewer) job opportunities and higher (lower) wage incomes. In addition, the increase in living cost can significantly reduce the desire of migrants, but it has not formed a push force for the local population to flee the city. If the housing price-to-income ratio of a city increases by 1%, its inflows will decrease significantly by 0.05%, but the outflows will also decrease significantly by 0.12%. This may be because a higher house price-to-income ratio often means a higher economic development level, facilitating the retention of local people in the city.

The migration stock has a significant positive effect on intercity migration with the highest regression coefficient. If the migration stock increases by 1%, the migration flows will increase significantly by 0.94%, indicating that the closer the social relationship, the greater the population migration. Migrants can establish social networks through family, friendships, colleagues and geo-relationship, promoting more migrants by providing them with help such as employment information, housing and transportation guidance. If the number of teachers per primary and secondary student, the number of tertiary hospitals and the number of books in the public library per 100 people increase by 1%, their inflows will increase by 0.04%, increase by 0.02% and drop by 0.009%, respectively, and their outflows will increase by 0.23%, reduce by 0.04% and reduce by 0.30%, respectively. This means the improvement of education and medical care can enhance the city's attractiveness, but cultural facilities cannot. Medical and cultural facilities can retain local people and reduce emigration, but education improvement can accelerate population exodus.

Population size has a significant positive effect on intercity population migration. If population size increases by 1%, its inflows and outflows will increase significantly by 0.01 and 0.88%, respectively, showing obvious asymmetry, meaning the influence of population size is dominated by push force. The impact of geographic distance doesn't meet expectations, but temporal distance has a significant negative impact. If the geographic distance and temporal distance increase by 1%, its migration flows will increase significantly by 0.02% and reduce significantly by 0.03%, respectively, indicating that the greater the geographic distance and the shorter the temporal distance, the greater the inter-city migration flows. This is because migrants do not choose the nearest cities as destinations but prefer cities that are provincial capitals and above with a higher economic development level. With the improvement of high-speed railways and airports, traditional spatio-temporal distance has been greatly compressed, enabling long-distance migration. The hindering effect of geographic distance is gradually weakening, while the friction effect of temporal distance remains significant.

#### 4.3.3. Network autocorrelation

Significant “destination-based,” “origin-based” and “origin-to-destination-based” spatial autocorrelation or spatial dependence relations are observed because ρ_*d*_, ρ_*o*_ and ρ_*w*_ are not significantly equal to 0. Among them, ρ_*d*_ and ρ_*o*_ are significantly >0, indicating a positive multilateral spillover effect. The migration flows from the same city tend to gather in a certain destination city and its surrounding cities. The migration flows moving to the same city also tend to come from a certain origin city and its surrounding cities, reflecting the spatial emulation behavior of migration flows. However, ρ_*w*_ is significantly < 0, indicating a negative multilateral spillover effect. The flows from the origin city to the destination city will inhibit the flows from surrounding cities of origin to surrounding cities of destination, reflecting the spatial competition behavior of migration flows. Notably, the spatial competition effect of migration flows is too small (ρ_*w*_= −0.0029) and even negligible, thus, the spatial emulation effect is dominant, emphasizing the important influence of social networks or path dependence on intercity population migration.

The Akaike info criterion (AIC) of the spatial econometric interaction model is smaller than that of the gravity model (AIC_spatialODmodel_ = 12,034.56 < AIC_gravitymodel_ = 12,633.53), implying that compared with the traditional gravity model, the spatial econometric interaction model not only considers the spatial dependence between flows, but also improves the fitting level of the model. In addition, the absolute values of the estimated coefficients of the spatial econometric interaction model are generally smaller than the gravity model, implying that the role of factors on population migration is often exaggerated when network autocorrelation is not considered.

## 5. Discussion

### 5.1. Economically developed cities remain the main destinations for population migration

Consistent with the results of existing studies, intercity population migration still tends to cluster in a few economically developed cities, especially the core cities of developed urban agglomerations, forming a “three big and two small” polycentric spatial pattern. The three big centers refer to the Beijing–Tianjin–Hebei, the Yangtze River Delta and the Pearl River Delta region, and the two small centers refer to the Chengdu–Chongqing City Cluster and the Triangle of Central China. The high-intensity migration flows occur mainly within the three major developed urban agglomerations in the eastern, moving from the peripheral cities to the core cities. Thus, the main direction of intercity migration flows is from the eastern to the eastern, which is different from the inter-provincial migration pattern. In central and western China, migration flows tend to gather in relatively developed provincial capital cities, such as Chengdu, Wuhan, Kunming, Xian and Urumqi.

This study also found that intercity population migration in China has the characteristics of hierarchical migration. Net migration flows go up the urban hierarchy, validating the economic law of migration to cities with high economic development levels again. However, it is a jump migration rather than Revenstein's step migration, with the largest inflows for any level being its exchanges with lower-middle level cities, rather than the next lower level. It is worth noting that net migration flows move upward the city hierarchy do not mean that all migration flows do as well; there are still a small number of downward migration flows that are likely to be related to environmental or amenity migration, especially for high-skilled and highly educated talent, they tend to have a tendency to flee from the high-class large cities because of unhealthy natural and social environment.

In addition, this case study from China has shown that destination cities with large number of migrants and high economic development levels are not necessarily the healthiest areas for the environment, which can be seen by the spatial pattern of environmental health factors. Environmentally friendly cities are mainly located in the southern region, and most of them are important origin cities for the floating population. While important destination cities for the floating population in developed eastern region tend to have relatively poor environmental conditions. Therefore, there is a spatial mismatch between the migrant gathering space and the good environmental space.

### 5.2. Environmental health factors have not yet become a determinant of population migration

Different from some existing research, this study found the effect of environmental health factors, such as air quality, climate comfort and ecological space, are inconsistent with expectations. Environmental health factors have not yet become a major consideration in migration decisions. However, socioeconomic factors remain the determinants in intercity migration, meaning that migrants pay more attention to economic needs rather than environmental health. This is because of China's development stage and the fact that most of China's floating population are low-skilled migrant workers who migrate more for economic purposes. On the one hand, different from developed countries, China is the largest developing country in the world. The development of industrialization exposes the cities themselves to many unhealthy environmental exposures, and migrants are willing to pay the price of environmental health to earn more money. On the other hand, the vast majority of the low-skilled migrant workers are poorly educated, have a heavy family livelihood burden, and do not have a high level of awareness of environmental health themselves. However, some studies have shown that highly educated and high-skilled migrants are increasingly concerned about environmental health (39, 62). They often have the ability to obtain satisfying jobs in large cities. On the basis of meeting basic economic needs, they are more concerned about air quality, climate comfort and other environmental health issues.

This study also found that except for economic factors such as wage income and job opportunities, social factors such as social networks, education and health care are also important influencing factors of intercity population migration. On the one hand, already settled migrants often act as a “bridgehead,” reducing the risks and costs of subsequent migration and settlement by providing information, organizing travel, finding jobs and housing and assisting in adaptation to a new environment, thus promoting more migration. The spatial emulation behavior among migration flows proves this path-dependent process. On the other hand, after satisfying basic material life needs, migrants also breed a demand for high levels of public service such as education and medical care. More and more migrants prioritize their quality of life over career prospects, especially for high-quality talents. In addition, the friction effect of geographic distance on intercity migration is not significant, while the friction effect of time distance is significant, indicating that migrants pay more attention to time distance rather than geographic distance.

Based on the existing literature and empirical studies in this paper, the internal mechanism of the impact of environmental health factors on population migration is summarized: Environmental health factors, together with socioeconomic factors, gravity factors and other factors, contribute to the process of population migration decisions and destination selection. When environmental changes threaten people's lives and property security, people will move passively, and their destination selections are more determined by the government or living conditions, such as ecological migrants or refugees. When environmental changes are not sufficient to threaten people's lives and property, people may choose not to migrate or to migrate voluntarily. For low-skilled migrant workers, the environmental health factors often do not play a decisive role. Although the environment may affect their health, they often make destination choices at the expense of environment quality to pursue stable employment and economic income. However, for high-skilled talents, the environmental health factors sometimes play a decisive role, poor environmental conditions may make them decide to leave or not to move in.

### 5.3. Policy implications

Owing to the hukou system, the floating population cannot enjoy the benefits and rights of permanent migrants and local residents in the destination city, such as social securities, health care and education opportunities. Thus, migrants face social vulnerability in the destination cities. Recently, the government has been aware of this problem and has relaxed hukou restrictions in large cities to actively promote the citizenship of the agricultural transfer population and the equalization of basic public services. However, the environmental health vulnerability of migrants has long been overlooked. Migration is generally followed by behavioral, lifestyle and environmental changes that can significantly increase the risk of disease in the early generations of migrants (63), and affect migrants' health. Thus, the government should pay attention not only to the social vulnerability of migrants, but also to their environmental health vulnerability.

The environmental health problems faced by China's population migration can provide policy implications for other developing and underdeveloped countries. Large cities with high immigration should pay more attention to environmental health issues and follow an environmentally friendly and sustainable development path in the process of urbanization, potentially increasing people's health wellbeing. Specifically, to focus on ecological construction, and expand ecological space such as parks, green spaces and forests. To strengthen environmental protection and governance, and reduce pollutant pollution. To promote green development, and develop circular economy and clean production.

This study also found that most migrants neither come from the poorest cities nor the poorest segments of the population. This is because migration involves significant costs and risks that the poorest generally cannot afford. This also means that people in poor cities benefit very little from the urbanization and migration process. Therefore, the government should pay more attention to the migration barriers of poverty areas and poverty population, and provide them with more labor export opportunities and migration cost subsidies. The government should also vigorously implement the rural revitalization strategy, increase industry cultivation and support and promote local urbanization.

## 6. Conclusion

Based on the 1‰ micro-database of the 2015 1% population sample survey, this study used the spatial visualization method and spatial econometric interaction model to examine the spatial patterns of intercity population migration and environmental health factors in China, and focus more on the impact of environmental health factors on intercity population migration. The conclusions are as follows.

First, the main direction of intercity population migration is still toward economically developed high class cities, especially the core cities of three major urban agglomerations in the eastern coast where the floating population is most active. However, these major destinations are not necessarily the healthiest areas for the environment. Second, environmentally friendly cities are mainly located in the southern region. The areas with less serious atmospheric pollution are mainly distributed in the south, climate comfort zones are mainly located in the southeastern region, and the areas with more urban green areas are mainly distributed in the northwestern region, all of which are not necessarily the main destination cities for floating populations. Third, compared with socioeconomic factors, environmental health factors have not yet become a major driver of population migration; migrants tend to place a higher value on income than on environmental health.

The contributions of this study are as follows. It found that economically developed cities are still the main destination for population migration. Then, it proved that environmental health factors have not yet become a determinant of population migration. This study also suggested the government should focus not only on the public service wellbeing of migrant workers but also on their environmental health vulnerability, contributing to the construction of a healthy city.

However, this study is not free from limitations. The first is that the data sample is biased. This study uses the 1‰ microdata, including a large number of zero flows, which does not mean that cities did not have migration flows but rather they were not collected when sampling. The second limitation is the selection of environmental health indicators. This study only selects three variables that are currently of most concern for the environmental health development in Chinese cities. In the future, more attention should be paid to the research on the relationship between more comprehensive environmental health factors and population migration based on individual migrant surveys.

## Data availability statement

Publicly available datasets were analyzed in this study. This data can be found at: https://microdata.stats.gov.cn/.

## Author contributions

PG was responsible for data processing, graph production, and paper writing and revision. WQ provided important insights and was responsible for paper revision. SL and ZL participated in paper revision. ZP was responsible for collecting and processing data. All authors contributed to the article and approved the submitted version.
